# A direct interaction between CENTLEIN and RABIN8 is required for primary cilium formation: CENTLEIN-RABIN8 interaction

**DOI:** 10.3724/abbs.2023064

**Published:** 2023-07-20

**Authors:** Liansheng Li, Junlin Li, Li Yuan

**Affiliations:** Savaid Medical School University of Chinese Academy of Sciences Beijing 101408 China

**Keywords:** CENTLEIN, RABIN8, CENTLEIN-RABIN8 interaction, pericentrosomal RABIN8, primary cilia

## Abstract

Primary cilia are formed in nearly all growth-arrested cells and are essential for mammalian development and tissue homeostasis. Defects in primary cilia result in a range of disorders in humans, named ciliopathies. The spatiotemporal localization of RABIN8 on the pericentrosome is an early step in ciliogenesis. Here, we show that CENTLEIN depletion causes the persistent accumulation of RABIN8 on the pericentrosome and primary cilium loss in hTERT-immortalized retinal pigment epithelial cells and murine embryonic fibroblasts. CENTLEIN interacts with RABIN8 directly. A stretch of a 31-amino acid sequence located in the 200‒230 region of the RABIN8 GEF domain is responsible for its physical interaction with CENTLEIN, while expression of the full-length but not the internal deletion lacking the RABIN8-binding site of CENTLEIN largely rescues the ciliogenesis defect provoked by CENTLEIN depletion. Expression of activated RAB8A partially reverses cilium loss in *CENTLEIN*-null RPE1 cells, so the functional importance of the CENTLEIN-RABIN8 interaction is defined.

## Introduction

The primary cilium (PC) present at the surface of most vertebrate cells [ 1, 2] is composed of a microtubule-based axoneme extending from the mother centriole [ 3– 5] and surrounded by the ciliary membrane studded with ciliary receptors [ 6– 9] and ion channels [5]. Because of its sophisticated features and wide distribution, the primary cilium thus acts as a signaling hub in diverse biological processes of tissue development. Defects in the formation or function of cilia lead to ciliopathies representing a range of complex human syndromes [ 10– 13]. PC formation takes place in quiescent cells, wherein a fully mature centriole can dock with the cell membrane to become a basal body that anchors a cilium [ 14– 16].

RAB11-dependent RABIN8 trafficking toward the centrosome is thought to be the earliest step in ciliogenesis [ 17– 19]. RAB8 GTPase, activated by the guanine nucleotide exchange factor (GEF) RABIN8, is the first membrane trafficking regulator shown to localize to the primary cilium and function in primary cilium formation [ 20– 23] and cholesterol trafficking to the plasma membrane [24]. During ciliogenesis, RAB11 traffics RABIN8 to the centrosome to activate RAB8 for its required function in ciliary growth [ 21, 22, 25]. This regulation of RABIN8 centrosomal trafficking is related to both Akt [26] and NDR2 kinases [27]. RABIN8 phosphorylated by Akt affects RAB8-dependent cilia assembly, while NDR2-mediated phosphorylation of RABIN8 is essential for ciliogenesis in PRE1 cells [27]. Although the list of ciliary proteins is growing, the precise composition and regulation of ciliogenesis still remain incompletely characterized [5].

In the present study, we report that CENTLEIN is required for primary cilium formation. CENTLEIN was previously identified as a microtubule-associated protein (MAP), and its overexpression protects microtubules (MTs) from cold- or nocodazole-induced depolymerisation [28]. More importantly, CENTLEIN acts as a molecular linker. In somatic cells, CENTLEIN was characterized as a centriolar protein mediating the interaction between C-Nap1 and CEP68 to maintain centrosome cohesion [29]. In mouse male germ cells, CENTLEIN mediates the interaction between SUN5 and PMFBP1 to maintain the integrity of the sperm head-to-tail coupling apparatus (HTCA). Inactivation of CENTLEIN in mice leads to sperm decapitation and male sterility [30]. This study reveals a novel role of CENTLEIN in ciliogenesis and presents its functional importance attributed to its direct interaction with RABIN8.

## Materials and Methods

### Plasmid construction

Full-length *CENTLEN* obtained from HeLa cDNA was cloned into the vector pCMV-MYC. The truncated mutants of *CENTLEN* were cloned into the vector pCMV-MYC. Full-length *RABIN8* was a gift from Johan Peranen (University of Helsinki, Helsinki, Finland) and was cloned into the pEGFP-C1 vector. Full-length and truncated mutants of *RABIN8* were cloned into the vector pGEX-4T-3. *pEGFP*- *RAB8A* and *RAB11* were purchased from Addgene (Watertown, USA). *CCDC11* was a gift from Li Wei (Guangzhou Women and Children’s Medical Center, Guangzhou, China). *SEC15*, *MCM7*, *CEP164*, *CP110*, *EB1*, *TFT20* , *DNM2*, *KIFC3* and *OCRL* were cloned into the vector pEGFP-C1 and obtained from HeLa cDNA.

### Antibodies

Mouse monoclonal antibody (clone 11A4) against the human N-terminal regions of CENTLEIN [amino acid (aa) residues 89–437] and rat monoclonal antibody (clone 9F8) against the human and mouse CENTLEIN (aa 1–280) mixture were generated by Absea Biotechnology Ltd. (Beijing, China). Mouse anti-MYC antibody (M192-3; MBL International, Woburn, USA), rabbit anti-GFP antibody (50430-2-AP; Proteintech, Chicago, USA), mouse anti-GST antibody (M20007L; Abmart, Shanghai, China), mouse anti-GAPDH antibody (AC033; ABclonal, Wuhan, China) were used at a 1:5000 dilution for western blot analysis. Mouse anti-γ-tubulin (1:1000; TU-30; sc51715; Santa Cruz, Santa Cruz, USA) was used for immunoprecipitation (IF). Rabbit anti-γ-tubulin mAb (1:1000; R26790) was obtained from ZEN-Bio (Chengdu, China); mouse anti-acetylated tubulin mAb (1:1000; Clone 6-11B-1) was obtained from Sigma (St Louis, USA); CEP164 (1:1000; 22227-1-AP) was obtained from Proteintech; mouse anti-PAX6 (1:500) and rabbit anti-SOX2 (1:1000) antibodies were provided by Jianwei Jiao (Institute of Zoology, Chinese Academy of Sciences, Beijing, China). The secondary antibodies were goat anti-mouse IgG (1:5000; ZB-2305; Zhong Shan Jin Qiao, Beijing, China), goat anti-rabbit IgG (1:5000; ZB-2301; Zhong Shan Jin Qiao), Alexa Fluor 594 goat anti-rat IgG (1:1500; A11007; Invitrogen, Carlsbad, USA), and Alexa Fluor 488 goat anti-mouse IgG (1:1500; A11006; Invitrogen).

### Cell culture and transfection

hTERT RPE-1 cells (CRL-4000; ATCC, Manassas, USA) were cultured in DMEM/F12 (1:1; HyClone, Logan, USA) supplemented with 10% fetal bovine serum (FBS; 10270106; Gibco, Carlsbad, USA), 0.01 mg/mL hygromycin B and 1% antibiotics/antimycotics at 37°C with 5% CO _2_. HEK293T cells (CRL-3216; ATCC) were cultured in DMEM (HyClone) supplemented with 10% FBS and 1% antibiotics/antimycotics with 5% CO _2_ at 37°C. To induce primary cilium formation in RPE-1 and MEF cells, the growth medium was replaced by serum-free medium for 48 h. Lipofectamine 2000 reagent (Invitrogen) was used for HEK293T cell transfection. Cells were analysed at 24 h after transfection.

### Animals

The *Centlein* ^‒/‒^ mice were generated as previously reported [30]. All animal experiments were performed according to the approved protocols from the Institutional Animal Care and Use Committee (IACUC) of the Institute of Zoology, Chinese Academy of Sciences, China (IOZ20170079).

### Isolation and culture of mouse embryonic fibroblasts (MEFs)

MEFs were isolated from mouse embryos at 12.5 days post coitum and cultured in DMEM (HyClone) supplemented with 10% FBS, 20 mM glutamine, and 1% antibiotics/antimycotics at 37°C with 5% CO _2_.

### siRNA and CRISPR/Cas9 experiments

For RNA-mediated interference, cells were transfected using Lipofectamine RNAiMAX (Invitrogen) according to the manufacturer’s protocol and harvested at 72 h post transfection. The siRNA sequences of *CENTLEIN* are 5′-GAGCTGAAGTACACGCAA-3′ and 5′-GTTGAAGTATCACAGAGTA-3′. The siRNA sequence of negative control is 5′-TTCTCCGAACGTGTCACGT-3′. The *CENTLEIN* RPE1 cells were generated by applying the CRISPR-Cas9 system using the pCAG-SpCas9-AeGFP plasmid. The *U6* promoter and the guiding sequence were added to the single-guide RNA (sgRNA) 5′-CCGGGAGCTGATAAAGAATTTGTA-3′ and 5′-CCGGTGAGATCTGGGTTTGTAACC-3′ on the pUC19-U6-sgRNA plasmid. Then, they were cotransfected using Lipofectamine 3000 (Invitrogen). Transfected RPE1 cells were sorted with the BD FACS Aria Fusion Cell Sorter (BD Biosciences, Franklin Lakes, USA) with GFP tunnel at 48 h post transfection, and single cells were seeded in a 96-well plate.

### Immunoprecipitation

Transfected HEK293T cells were lysed in ice-cold ELB buffer [50 mM HEPES (pH 7.4), containing 250 mM NaCl, 0.1% NP-40, 1 mM phenylmethanesulfonylfluoride (PMSF; P7626; Sigma), and complete EDTA-free protease inhibitor cocktail (04693132001; Roche, Basel, Switzerland)], and then centrifuged at 12,000 *g* for 10 min. The supernatant was precleared by incubation with 50 μL of protein G-Sepharose (CW0012A; Cowin Biotech, Taizhou, China) at 4°C for 3 h. Then, 2 μg antibody was added to the supernatant and incubated at 4°C for an additional 3 h. After that, 20 μL Dynabeads-protein-G (10004D; Invitrogen) were added into the supernatant and incubated at 4°C overnight. After six times wash with lysis buffer, the immunoprecipitates were heated at 99°C for 10 min with SDS loading buffer and subjected to western blot analysis.

### Western blot analysis

Proteins obtained from lysates or immunoprecipitants were separated by SDS-PAGE and transferred to polyvinylidene difluoride (PVDF) membranes (IPVH00010; Millipore, Billerica, USA). The membranes were blocked in 5% nonfat milk (diluted in TBS-T: 10 mM Tris-HCl pH 7.4, 150 mM NaCl, and 0.1% Tween-20) at room temperature for 1 h and incubated with primary and secondary antibodies. Finally, ECL prime western blotting detection reagent (RPN 2232; GE Healthcare-Life Sciences, Marlborough, USA) was used for membrane development. A Tanon 4100 imaging system (Shanghai, China) was used for image exposure with GelCap 5.6 software (Tanon, Shanghai, China).

### *In vitro* binding assays

GST and GST fusion proteins were expressed in *E*. *coli* strain BL21, and purified by affinity chromatography with glutathione Sepharose 4B (17-0757-01; GE Healthcare) and then crosslinked to Sepharose beads. Lysates of HEK293T cells that had been transiently transfected with MYC *-CENTLEIN* in ELB were incubated with GST or GST fusion proteins (1 mg) bound to the beads (20 mg) overnight at 4°C. After four times wash with ELB buffer, proteins were extracted from the Sepharose beads by boiling in SDS-PAGE sample buffer and were then analyzed by western blot analysis.

### Immunofluorescence, immunofluorescence on tissue sections and image analysis

For immunofluorescence, cold methanol or 4% paraformaldehyde (PFA) was used for fixation of cells on cover slips for 8 min or 10 min at room temperature (RT), respectively. Then, cells were treated with 0.2% Triton X-100 for 5 min, rinsed with PBS three times, and blocked with 5% bovine serum albumin (BSA; AP0027; Amresco, Washington, USA) for 40 min. The primary antibody was added to the cover slips and incubated at 4°C overnight, followed by incubation with secondary antibody. The nuclei were stained with DAPI (D3571; Life Technologies, Carlsbad, USA). E10.5 mouse embryos were dissected and washed with PBS. Tissue samples were fixed with 4% paraformaldehyde for 20 min and then exchanged with 15% sucrose for 3 h and 30% sucrose overnight at 4°C. Tissue-Tek O.C.T. Compound (4583; Sakura, Oakland, USA) was used to embed the tissue samples. The tissue samples were subsequently stored at ‒80°C. Sections of 10 μm were made using a Leica 1950 cryostat (Leica, Wetzlar, Germany) and stored at ‒80°C. The sections were recovered at RT, rinsed with PBS, permeabilized in 0.2% Triton X-100 for 6 min, and washed with PBS for 5 min. Then, 5% BSA was used to block the prepared sections for 40 min at RT, and the sections were incubated with primary antibody overnight at 4°C. The sections were washed 3 times for 10 min with PBS and subsequently incubated with secondary antibodies (1:1500) for 1 h at RT. DNA was stained with DAPI (D3571; Life Technologies) for 10 min. The IF images were captured using an SP8 microscope equipped with a 63× oil immersion objective. The imaging software of SP8 microscopes is Leica Application Suite X (LASX) 3.0. IMARIS 9.8.2 was used for exporting images and further analysis.

### Statistical analysis

As previously reported [30], the statistical significance of the differences between the mean values of the different groups was measured by Student’s *t* test with paired, two-tailed distributions. Statistical analysis was performed by using the GraphPad Prism 7 and Microsoft Excel 2010 software. The differences were considered statistically significant when *P*<0.05.

## Results

### *CENTLEIN* depletion impairs primary cilium formation

**Figure 1:**
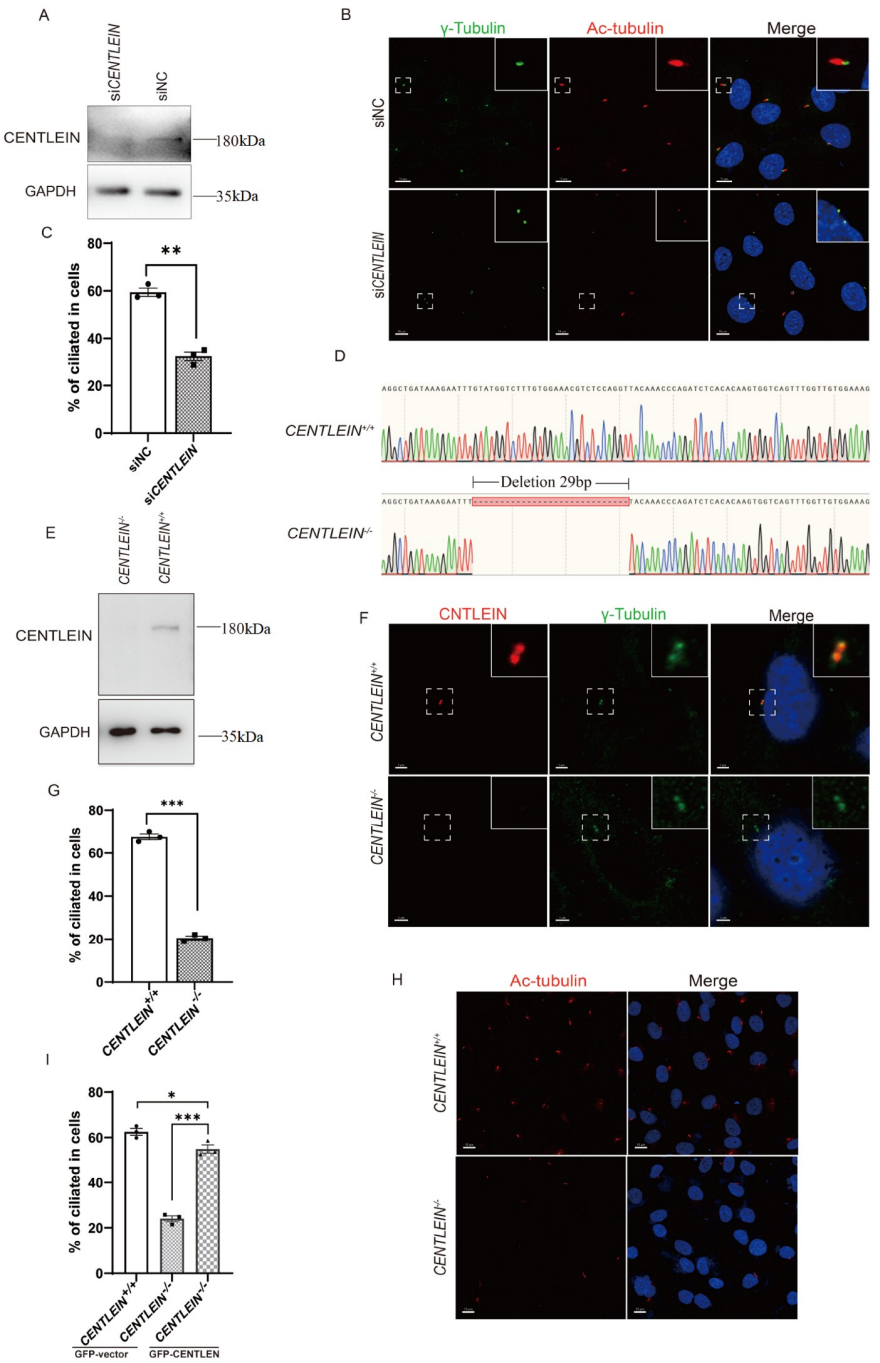
Figure 1 Knockdown and knockout of *CENTLEIN* in hTERT RPE1 cells (A) Western blot analysis of negative control and CENTLEIN knockdown. RPE1 cells were transfected with negative control or CENTLEIN siRNAs (30 nM) and cultured for 72 h. Cell lysates were analysed by immunoblotting with 11A4. (B) Effect of CENTLEIN depletion on ciliogenesis. RPE1 cells transfected with siRNAs (30 nM) were cultured for 48 h and serum-starved for 48 h. Cells were stained for γ-tubulin (green), Ac-tubulin (red) and DAPI (blue). Scale bar: 10 μm. Insets show magnifications of the boxed areas. Scale bar: 4 μm. (C) Quantification of siNC (59.460%±1.728%) and siCENTLEIN (32.390%±1.915%) ciliated cells. n=3; >200 cells per experiment; **P<0.01. (D) Schematic represents the designed target site; the sequence peak map displays a 29 bp deletion in exon 2. (E) Western blot analysis of CENTLEIN+/+ and CENTLEIN‒/‒ cell line lysates using 11A4. (F) Cells were costained with antibodies against CENTLEIN (red), γ-tubulin (green) and DAPI (blue). The insets show enlarged views of centrosomes. Scale bar: 3 μm; 0.5 μm (insets). (G,H) Effect of CENTLEIN knockout on ciliogenesis. (G) Quantification of ciliated cells in the CENTLEIN+/+ (67.460%±1.316%) and CENTLEIN‒/‒ (20.490%±0.914%) groups. n=3; >200 cells per experiment; ***P<0.001. (H) CENTLEIN+/+ and CENTLEIN ‒/‒ cell lines were serum-starved for 48 h. Cells were stained for Ac-tubulin (red) and DAPI (blue). Scale bar: 15 μm. (I) Quantification of CENTELIN+/+ and CENTLEIN ‒/‒ ciliated cells transfected with GFP-vector (62.470%±1.529%), GFP-vector (24.000%±1.329%) and GFP-CENTLEIN (54.920%±1.850%), n=3; >200 cells per experiment; *P<0.05, ***P <0.001.

In the course of characterization of CENTLEIN, we observed that siRNA-mediated *CENTLEIN* depletion impaired ciliation in RPE1 cells ( Figure 1A,B). To verify this finding, we used CRISPR/Cas9-mediated gene editing in RPE1 cells to ablate *CENTLEIN*. We isolated clones with frame-shift mutations in exon 2 of *CENTLEIN* ( Figure 1D), and the cell line was devoid of CENTLEIN protein ( Figure 1E,F). To rule out off-target effects, we showed that the expression of GFP-tagged CENTLEIN in these cells largely rescued the ciliogenesis defect ( Figure 1I). *CENTLEIN*-sufficient RPE1 cells displayed high levels of ciliogenesis upon serum withdrawal, with 67.46%±1.316% of cells carrying primary cilia, but ciliation was reduced by 47% in *CENTLEIN*-deficient cells ( Figure 1G,H), which was consistent with our previous observation ( Figure 1B,C).

**Figure 2:**
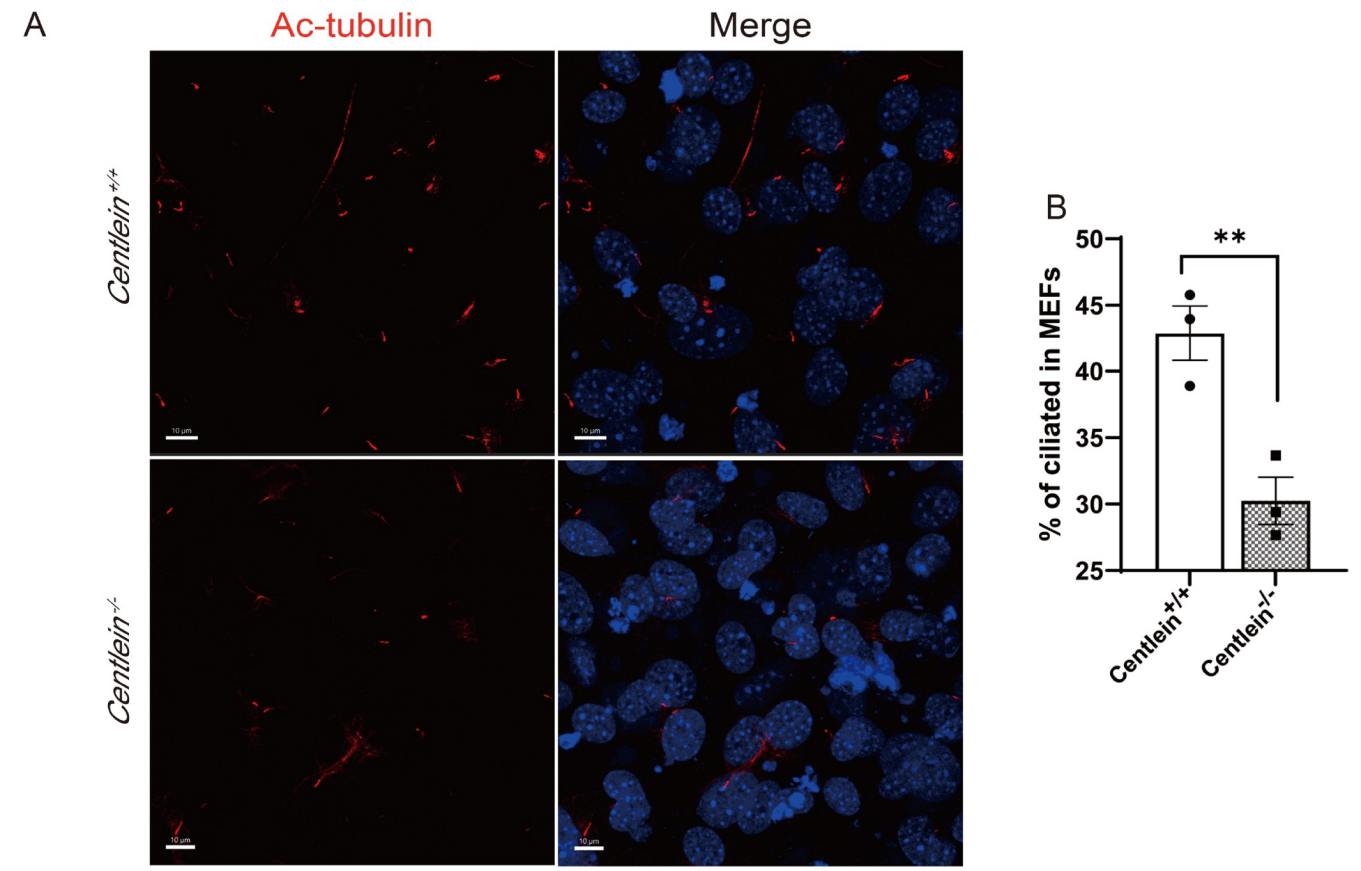
Figure 2 The influence of *CENTLEIN* depletion on cilia in mice The MEFs of Centlein+/+ and Centlein‒/‒ were serum-starved for 48 h. (A) Cells were stained for Ac-tubulin (red) and DAPI (blue). Scale bar: 10 μm. (B) Quantification of Centlein +/+ (42.880%±2.064%) and Centlein‒/‒ (30.25%±1.72%) ciliated MEFs. n=3; >200 cells per experiment; **P<0.001.

To further evaluate the effect of CENTLEIN on primary cilia, MEFs were isolated from wild-type and *Centlein*-null embryos at E12.5 [30]. Stained with anti-acetylated α-tubulin, *Centlein* ^‒/‒^ MEFs displayed a significantly reduced number of ciliated cells compared to *Centlein* ^+/+^ MEFs ( Figure 2). We thus conclude that CENTLEIN is required for primary cilium formation.

### CENTLEIN interacts with KIFC3 and RABIN8

**Figure 3:**
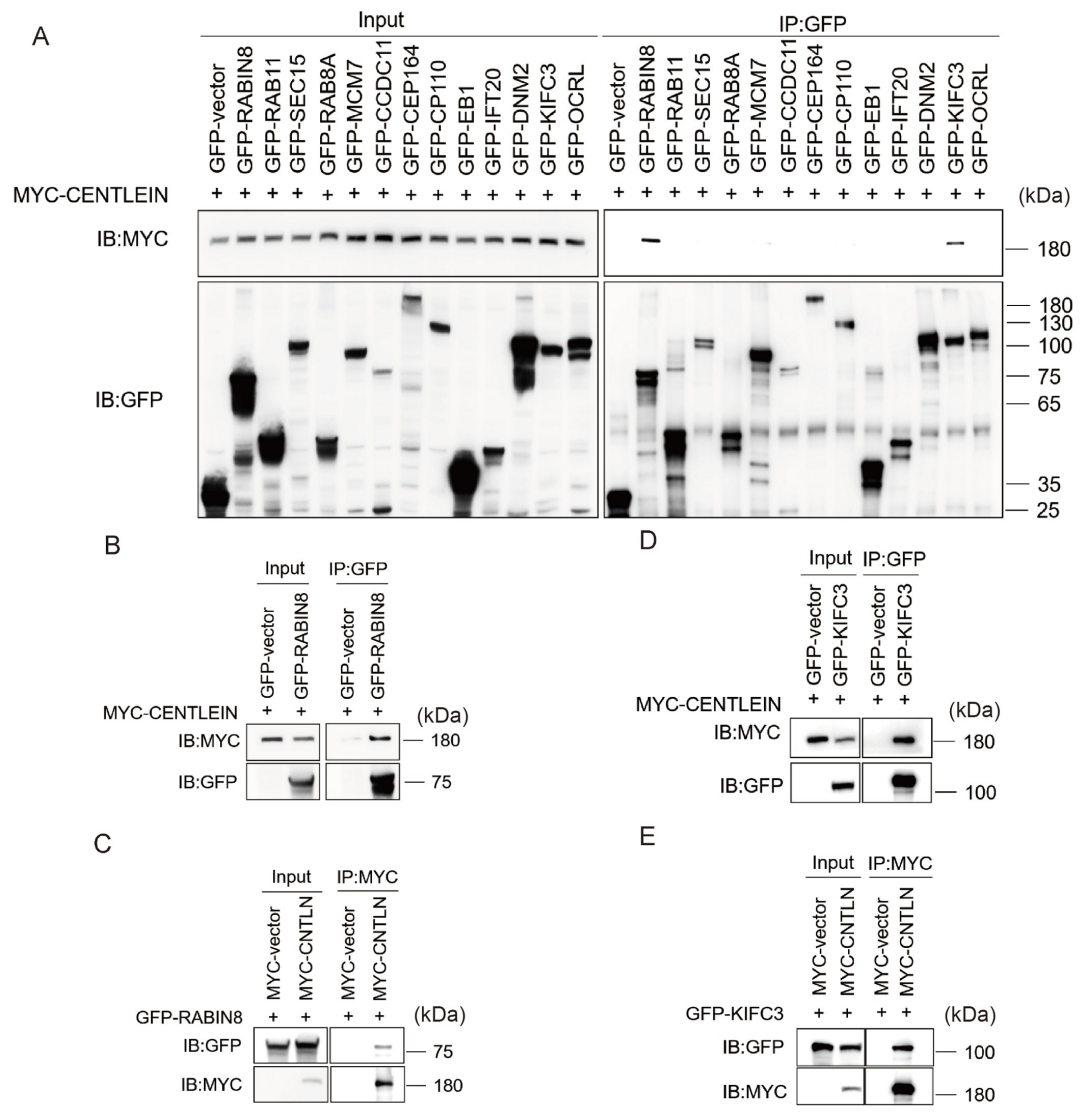
Figure 3 CENTLEIN interacts with RABIN8 and KIFC3 (A) A candidate-based approach used for the identification of CENTLEIN binding proteins. HEK293T cells were transfected with MYC-CENTLEIN and one of the GFP-tagged plasmids, including the empty vector, GFP-RABIN8, GFP-RAB11, GFP-SEC15, GFP-RAB8A, GFP-MCM7, GFP-CCDC11, GFP-CEP164, GFP-CP110, GFP-EB1, GFP-IFT20, GFP-DNM2, GFP-KIFC3 and GFP-OCRL for 48 h, immunoprecipitated (IP) with anti-GFP antibody and immunoblotted with MYC and GFP antibodies. (B,C) CENTLEIN could bind with RABIN8. MYC-CENTLEIN was cotransfected with either GFP-vector or GFP-RABIN8 into HEK293T cells for 48 h. Anti-GFP (B) or anti-MYC (C) immunoprecipitations were performed and analysed by western blot analysis using anti-GFP antibody and anti-MYC antibody. (D,E) CENTLEIN could bind with KIFC3. MYC-CENTLEIN was cotransfected with either GFP-vector or GFP-KIFC3 into HEK293T cells for 48 h. Anti-GFP (D) or anti-MYC (E) immunoprecipitations were performed and analysed by western blot analysis using anti-GFP antibody and anti-MYC antibody, respectively.

To gain an insight into the molecular mechanism underlying the ciliogenesis defect caused by CENTLEIN depletion, 13 centrosome- and/or cilium-related proteins were selected as the first batch of proteins screened for their interaction with CENTLEIN. To this end, we cotransfected MYC-tagged CENTLEIN and a GFP-tagged plasmid encoding a cilium or centrosome protein into HEK293T cells and then used an anti-MYC antibody to blot anti-GFP immunoprecipitate. As shown in Figure 3A, only KIFC3 and RABIN8 were present in the MYC-CENTLEIN immunoprecipitate.

**Figure 4:**
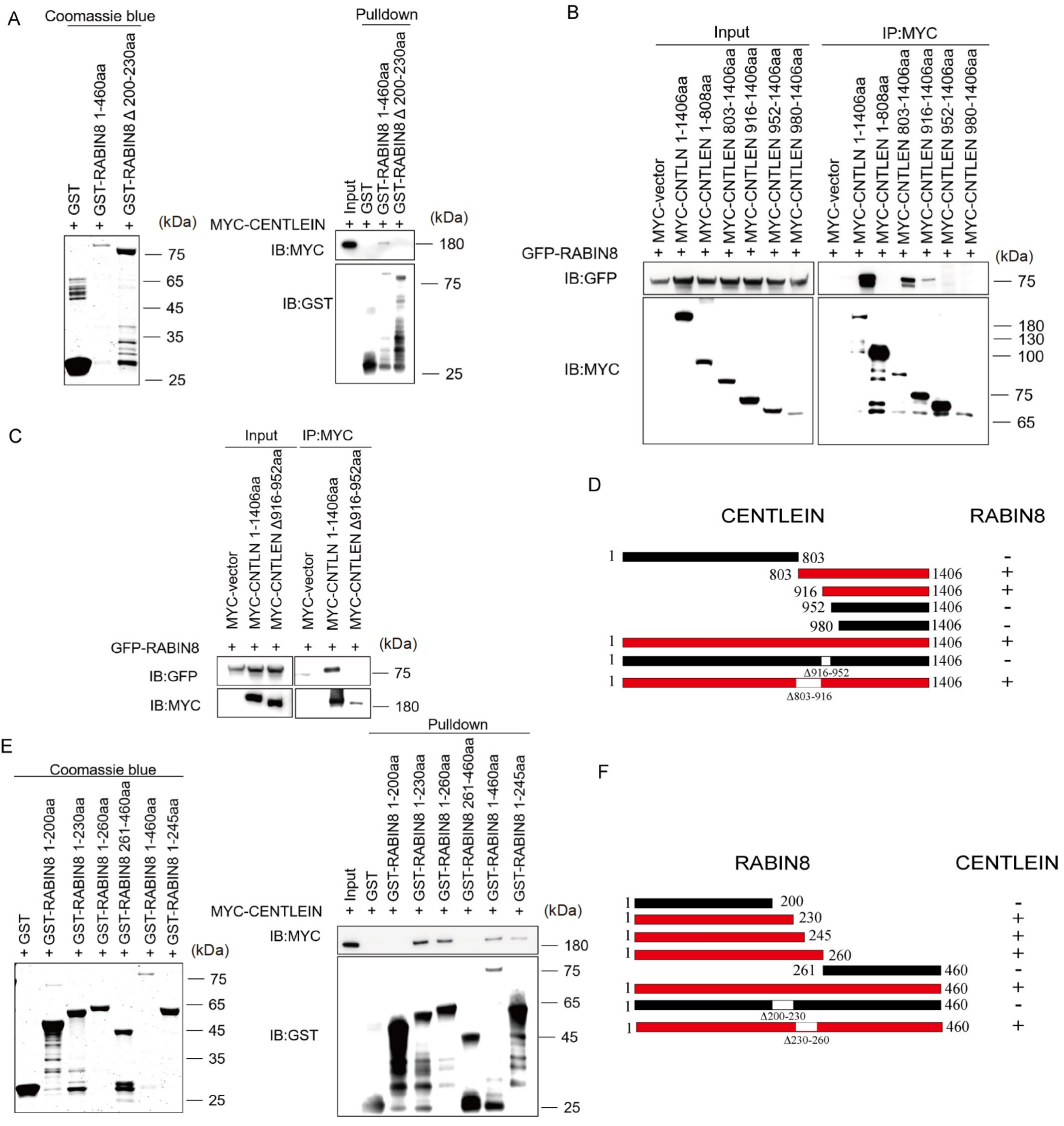
Figure 4 CENTLEIN interacts with RABIN8 directly (A) Amino acids 1‒460 and depletion of 200‒230 (Δ200‒230) of RABIN8 with GST-tag were extracted from bacteria BL21 and purified and then used to pull down MYC-CENTLEIN from HEK293T lysate. (B‒D) Amino acids 916‒952 of CENTLEIN are essential for its binding with RABIN8. HEK293T cells were cotransfected with GFP-RABIN8 and the indicated fragments of MYC-CENTLEIN, and anti-MYC immunoprecipitations were performed and analyzed by western blot analysis using anti-GFP antibody and anti-MYC antibody, respectively (B). GFP-RABIN8 was cotransfected with either MYC-CENTLEIN 1‒1406 or MYC-CENTLEIN without 916‒952 (Δ916‒952) into HEK293T cells for 48 h, and anti-MYC immunoprecipitations were performed and analysed by western blot analysis using anti-GFP antibody and anti-MYC antibody, respectively (C). +, red, interaction; ‒, black, no interaction (D). (E) Amino acids 200‒260 of RABIN8 are necessary to for its binding with CENTLEIN. Amino acids 1‒200, 1‒230, 1‒260, 261‒460, 1‒460 and 1‒245 of RABIN8 with a GST tag were extracted from bacteria BL21, purified, and then used to pull down MYC-CENTLEIN from HEK293T lysates. (F) Amino acids 200‒230 of RABIN8 directly bind to CENTLEIN, +, red, interaction; ‒, black, no interaction.

RABIN8 (also known as Rab3ip), the guanine nucleotide exchange factor for RAB8, is delivered to the centrosome on vesicles to activate RAB8 to promote ciliary membrane assembly [ 3, 18 , 20, 31]. We therefore confirmed the CENTLEIN-RABIN8 interaction by reciprocal coimmunoprecipitation assays. MYC-CENTLEIN was specifically coimmunoprecipitated with GFP-RABIN8 ( Figure 3B), and in a reverse direction, RABIN8 could readily be detected in the MYC-CENTLEIN immunoprecipitate ( Figure 3C). The CENTLEIN-KIFC3 interaction was also verified by reciprocal immunoprecipitation/blotting experiments ( Figure 3D,E). Follow-up GST pull-down experiments were carried out. GST-RABIN8 and GST proteins were incubated with lysates from HEK293 cells expressing MYC-CENTLEIN and immobilized on glutathione beads. CENTLEIN was pulled down by GST-RABIN8 ( Figure 4A) but not by GST ( Figure 4A, right), indicating that CENTLEIN interacts with RABIN8 directly.

We next performed domain-mapping studies to identify the region(s) of CENTLEIN responsible for its interaction with RABIN8, and vice versa. We transfected HEK293T cells with MYC-tagged deletion constructs of CENTLEIN, performed anti-MYC immunoprecipitations ( Figure 4B), and defined the RABIN8-binding region between residues 916 and 952 ( Figure 4C,D and Supplementary Figure S1B). Deletion analysis of RABIN8 revealed that aa 200–230 located in the RABIN8 GEF domain was crucial for RABIN8 binding with CENTLEIN ( Figure 4E,F and Supplementary Figure S1A) because the fusion proteins deleted of this sequence did not bind with CENTLEIN ( Figure 4A), whereas all proteins containing this sequence could bind with CENTLEIN ( Figure 4E). Altogether, these data indicate that the interaction between CENTLEIN and RABIN8 is mediated by fewer than 3-dozen amino acids of each protein.

### *CENTLEIN* depletion causes the persistent accumulation of RABIN8 on the pericentrosome

**Figure 5:**
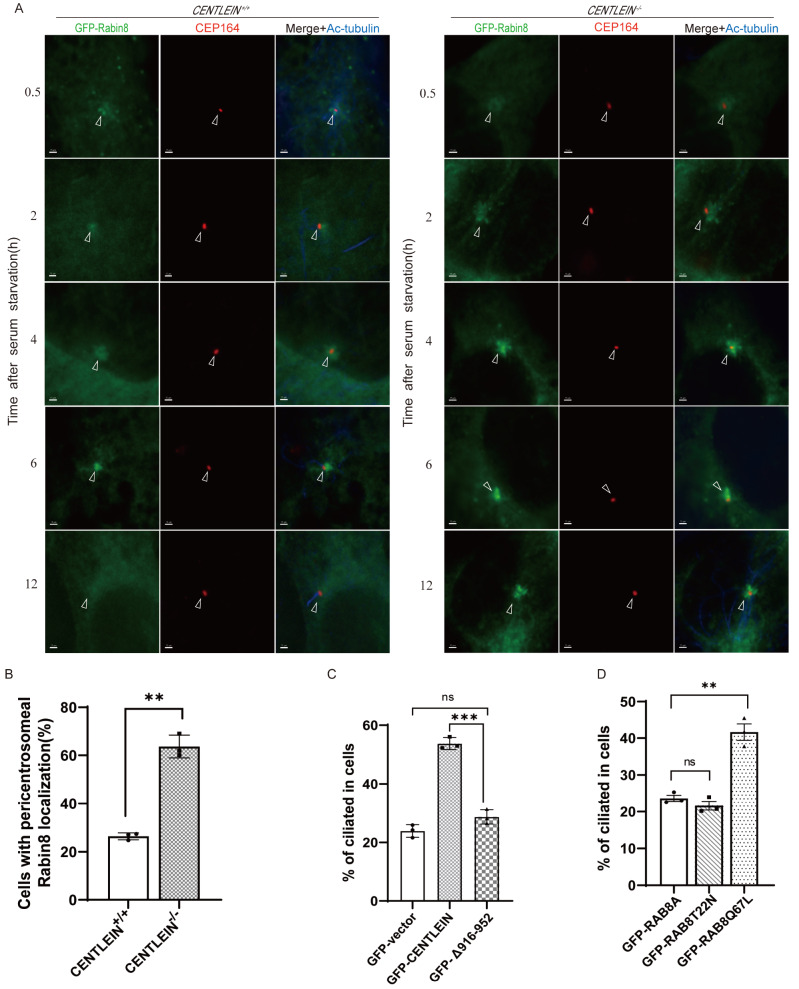
Figure 5 RABIN8 and RAB8 affect cilia in *CENTLEIN* ^‒/‒^ cells (A) Time course of RABIN8 localization after serum starvation. CENTLEIN+/+ and CENTELIN–/– RPE1 cells stably expressing GFP-RABIN8 were serum-starved. At the indicated time points, cells were fixed and imaged by GFP fluorescence (green), basal body marker of centrosome CEP164 (red) and Ac-tubulin (blue) immunostaining. Arrowheads indicate the position of the centrosome. Scale bar: 15 μm. (B) Quantification of the number of cells with pericentrosomal localization of GFP-Rabin8 at 12 h after serum starvation in CENTLEIN+/+ (26.420%±0.826%) and CENTLEIN‒/‒ (63.660%±2.702%) cells. n=3; >200 cells per experiment; **P<0.01. (C) Quantification of CENTLEIN-/- ciliated cells transfected with GFP-vector (23.920%±1.247%), GFP-CENTLEIN (53.770%±1.178%) and GFP-Δ916‒952 (28.710%±1.417%). n=3; >200 cells per experiment; *** P<0.001. (D) Quantification of CENTLEIN‒/‒ ciliated cells transfected with GFP-RAB8A (23.65%±0.84%), GFP-RABT22N (21.63%±1.19%) and GFP-RAB8Q67L (41.63%±2.22%). n=3; >200 cells per experiment; **P<0.01.

An early step in ciliogenesis is RABIN8 trafficking to the centrosome in a RAB11-dependent manner [17]. Upon serum withdrawal, GFP-RABIN8 rapidly traffics to the centrosome and transiently accumulates on the pericentrosome within 0.5 h ( Figure 5A; 0.5 h). Within 6 h of serum starvation, the cells with pericentrosomal localization of GFP‐RABIN8 accumulated [ 17, 27] ( Figure 5A; 2‒6 h). As the number of ciliated cells stained with acetylated tubulin (a marker for primary cilia) was gradually increased, the pericentrosomal localization of GFP-RABIN8 dispersed diffusely throughout the cytoplasm ( Figure 5A).

To determine the significance of the CENTLEIN-RABIN8 interaction *in vivo*, CENTLEIN-deficient and CENTLEIN-sufficient RPE1 cells stably expressing GFP-RABIN8 were examined 12 h after serum deprivation. At that time point, GFP-RABIN8 in CENTLEIN-sufficient cells was dispersed diffusely throughout the cytoplasm in 74% of cells, but GFP-RABIN8 in CENTLEIN-deficient cells was largely retained on pericentrosomal vesicles ( Figure 5A,B; 12 h). This aberrant accumulation suggests that CENTLEIN is required for the spatiotemporal localization of RABIN8 on the pericentrosome.

### Reciprocal binding sites of CENTLEIN and RABIN8 define their ciliary function

To establish the functional importance of the RABIN8-binding region within CENTLEIN, we performed rescue experiments. Expression of GFP-tagged CENTLEIN (WT), but not the internal deletion CENTLEIN (Δ916‒952) in CENTLEIN-null cells, largely rescued the ciliogenesis defect (GFP-CENTLEIN, 53.77%±1.178%; GFP-Δ916‒952, 28.71%±1.417%) ( Figure 5C).

Given the RABIN8 binding site for CENTLEIN located in the GEF domain whose activity towards RAB8 is essential for ciliogenesis [ 17, 22, 31 , 32], we transfected *CENTLEIN*-null cells with GFP-tagged RAB8A (WT), its constitutively active RAB8A (Q67L) and dominant-negative RAB8A (T22N) mutant. Both RAB8A (Q67L) and RAB8A (T22N) are often used to perturb the GTP/GDP cycle of small G proteins. The T22N mutation maintains the protein in the GDP-bound state and binds guanine exchange factors to inhibit their activity on native substrates, whereas the Q67L mutation reduces GTP hydrolysis and keeps the protein in the GTP-bound state. As shown in Figure 5D, 42% of RAB8Q67L-expressing *CENTLEIN*-null cells developed cilia, comparable to ciliation levels observed in *CENTLEIN*-null cells expressing either RAB8A (WT) or Rab8T22N (wild type, 23.7%±0.89%; T22N, 21.5%±1.2%. Partial reversion of cilium loss in *CENTLEIN*-null cells by Rab8Q67L indicates that the activated RAB8A counteracts the *CENTLEIN*-null effect on ciliogenesis. Taken together, the functional significance of the CENTLEIN-RABIN8 interaction is attributed to their binding sites.

### Ciliary density in the ventral neural tube is unaltered upon *CENTLEIN* depletion

**Figure 6:**
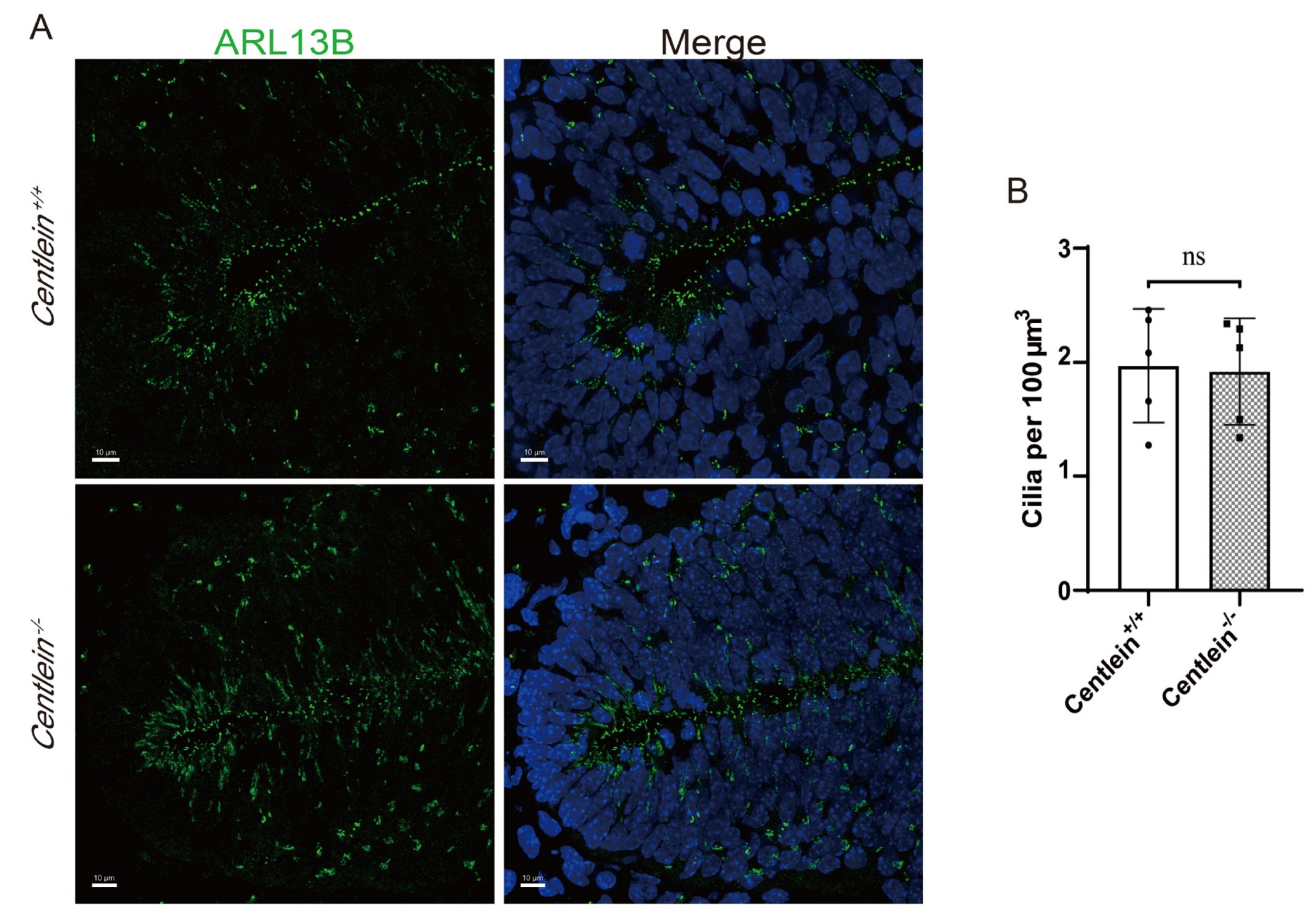
Figure 6 *Centlein*-null effect of cilia on E10.5 ventral neural tube (A) Centlein +/+ and Centlein‒/‒ E10.5 ventral neural tubes stained for ARL13B (green) and DAPI (blue). Scale bar: 10 μm. (B) Quantification of Centlein+/+ (1.97%±0.22%) and Centlein‒/‒ (1.92%±0.21%) ventral neural tube cilia per 100 μm3. n=5; P=0.8755.

Finally, the effect of *CENTLEIN* depletion on ciliogenesis in the ventral neural tube of mouse embryos was assessed. Labelled with the cilia-specific ARL13B antibody, the number of cilia within the known volume of the ventral neural tube was determined by using 3D image stacks from confocal images [33]. Cilia density in the ventral neural tubes of E10.5 *Centlein* ^‒/‒^ embryos was indistinguishable from that in their *Centlein* ^+/+^ littermates ( Figure 6A,B). In addition, the expression of Pax6 ( Supplementary Figure S2A), a dorsal marker and SOX2 ( Supplementary Figure S2B) representing the status of neural progenitors [34] was also unaffected in the *Centlein* ^‒/‒^ neural tube, suggesting that the sonic hedgehog (shh) pathway is not impaired. CENTLEIN is indispensable for primary cilia formation in the ventral neural tube.

## Discussion

In the present study we reveal a novel role of CENTLEIN in primary cilium formation in both RPE-1 cells and MEFs but not in the ventral neural tube of mouse embryos. Patterning of the embryonic neural tube requires primary cilia, on which several signaling pathways, especially Shh, depend. During the development of the nervous system, Shh activity is crucial to control the proliferation and dorso-ventral patterning of the neural tube [35]. The Shh signaling pathway is tightly linked to the primary cilium [36]. In the developing neural tube, a decreasing ventral-to-dorsal Shh gradient regulates the expressions of various genes encoding transcription factors [37]. The subsequent antagonistic interactions among these transcription factors (FoxA2, NKX6.1, PAX6 and PAX7) sharpen gene expression boundaries in the neural tube and promote neuronal differentiation [38]. In vertebrates, most of the Hedgehog (Hh) signaling pathway components are located at or in the proximity of the primary cilium [35]. The Shh pathway is activated when the receptor Ptch1 binds to Shh [39], which causes Ptch1 to exit the cilium and Smoothened (Smo), the pathway transductor, to enter [40]. Smo accumulation prevents GLI processing (mainly GLI3R) and leads to the generation of GLI activator forms (mainly GLI2A) [41], albeit through unknown mechanisms. In the *centlein*-null developing neural tube, the Shh signaling pathway appears to be normal.

We have previously reported that CENTLEIN, as a newly identified microtubule-associated protein (MAP), directly binds to purified microtubules (MTs) via its longest coiled-coil domain, and its overexpression results in profound nocodazole- and cold-resistant MT bundles, which also rely on its MT-binding domain [28]. We have now found that CENTLEIN physically interacts with RABIN8 and that its depletion causes the persistent accumulation of RABIN8 on the pericentrosome. In 2019, Cuenca *et al* . [42] reported that the microtubule-associated protein 11 (MAP11, also known as C7orf43/TRAPPC14) directly bound to RABIN8 and that its knockdown diminished RABIN8 preciliary centrosome accumulation and, in turn, affected ciliogenesis in both human cells and zebrafish embryos. Both CENTLEIN and C7orf43 are RABIN8-binding MAPs and are required for ciliogenesis. Of note, the ablation of the former causes the persistent accumulation of RABIN8 on the pericentrosome, whereas the depletion of the latter reduces RABIN8 focal accumulation in the centrosomal region [42].

The primary cilium is mainly composed of axonemal microtubules elongating from the distal end of the basal body and a ciliary membrane surrounding the axoneme, accessorized by motor proteins and intraflagellar transport (IFT) particles [ 43– 45]. Transport in and out of the cilium, which is necessary for ciliogenesis, maintenance and signaling, occurs bidirectionally along microtubules via the process of intraflagellar transport [46]. It is well established that the dynamics and organization of the MT cytoskeleton are regulated largely by MAPs [ 47‒ 50]. Given the critical roles of MAPs in maintaining and regulating MT stability [ 51, 52] and intracellular transport [50], the current work adds one more piece to the puzzle of how the spatiotemporal localization of RABIN8 on the pericentrosome is controlled, while important questions remain to be answered.

In summary, besides its vital roles in the maintenance of sperm head-tail integrity [30] and centrosome cohesion [29], CENTLEIN is required for primary cilium formation. CENTLEIN physically interacts with RABIN8, in which the 31 amino acid sequence located at 200‒230 aa of the RABIN8 GEF domain [ 17, 20, 27] is responsible for the interaction. Expression of epitope-tagged CENTLEIN (WT), but not the internal deletion CENTLEIN (Δ916‒952) lacking the RABIN8-binding site, largely rescued the ciliogenesis defect provoked by CENTLEIN depletion. Notably, the RABIN8-binding site separates from the centrosome-targeting domain ( Supplementary Figure S3). CENTLEIN ablation causes the persistent accumulation of RABIN8 on the pericentrosome, while expression of activated RAB8A partially reverses cilium loss in *CENTLEIN*-null RPE1 cells. Future studies will focus on elucidating the underlying mechanism(s) of RABIN8 release from the pericentrosome as cilium formation proceeds.

## Supporting information

22722supplementary_figures

## Supplementary Data

Supplementary data is available at *Acta Biochimica et Biophysica Sinica* online.
