# Supplementary material for: A direct interaction between CENTLEIN and RABIN8 is required for primary cilium formation: CENTLEIN-RABIN8 interaction
Source: Acta Biochim Biophys Sin (Shanghai). 2023 Jul 20;55(9):1434–44. doi: 10.3724/abbs.2023064 (PMC10520482; doi:10.3724/abbs.2023064)
Supplement: 22722supplementary_figures [file 22722supplementary_figures.pdf]

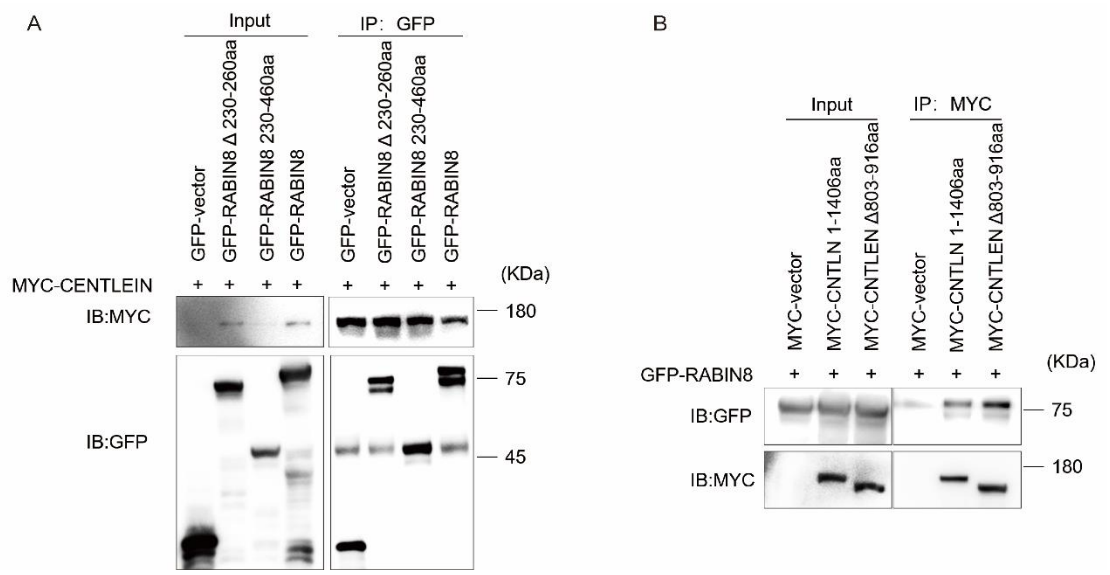

Supplementary Figure S1. CENTLEIN interacts directly with RABIN8 (A) HEK293T cells were co-transfected with MYC-CENTLEIN and the indicated fragments of GFP-RABIN8 (Δ230–260, 230–460 and 1–460), anti-GFP immunoprecipitations were performed and analysed by western blot analysis using anti-GFP antibody and anti-MYC antibody, respectively. (B) HEK293T cells were co-transfected with GFP-RABIN8 and the indicated fragments of MYC-CENTLEIN (1–1406 and Δ803–916), anti-MYC immunoprecipitations were performed and analysed by western blot analysis using anti-GFP antibody and anti-MYC antibody, respectively.

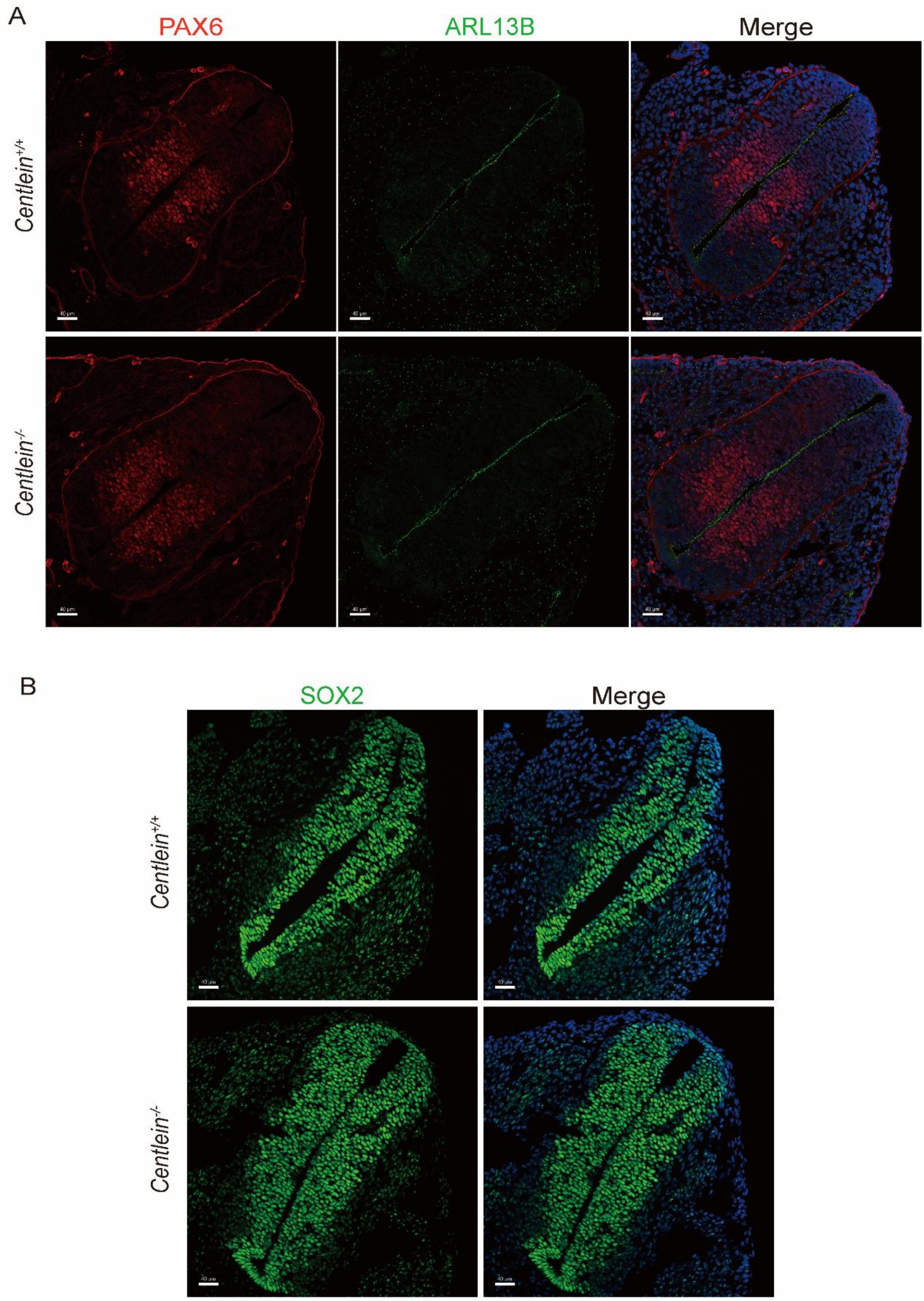

Supplementary Figure S2. Effect of CENTELIN depletion on E10.5 mouse neural tube (A) *Centlein*$^{+/+}$ and *Centlein*$^{−/−}$ E10.5 neural tube stained for PAX6 (red), ARL13B (green) and DAPI (blue). Scale bar: 40 μm. (B) *Centlein*$^{+/+}$ and *Centlein*$^{/−}$ E10.5 neural tube stained for SOX2 (green) and DAPI (blue). Scale bar: 40 μm.

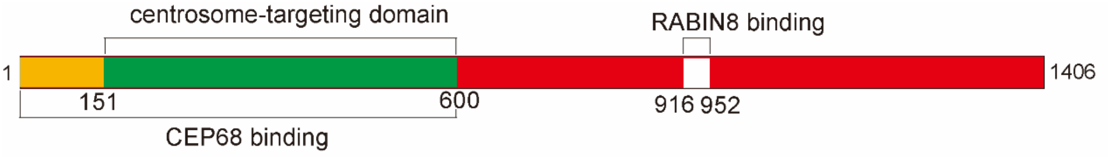

Supplementary Figure S3. Schematic diagram of the different domains of CENTLEIN for its RABIN8-binding and centrosome localization Centrosome-targeting domain (aa 151–600 binding to Centrosomal CEP250) is shown in green and RABIN8- binding domain (aa 916–952) is shown in white. Centrosomal CEP68-binding domain (aa 1–600) is also shown in orange and green.
